# Supplementary material for: Mapping the evolution of fertility support policies in China: A content and instrumental analysis
Source: PLoS One. 2025 Oct 9;20(10):e0332137. doi: 10.1371/journal.pone.0332137 (PMC12510515; doi:10.1371/journal.pone.0332137)
Supplement: S1 Appendix — (ZIP) [file pone.0332137.s001.zip › S1 Appendix. 226 original policy documents/179-中共中央办公厅、国务院办公厅关于转发《国务院妇女儿童工作委员会关于三年来我国妇女儿童工作情况和“九五”期间工作任务的报告》的通知(FBM-CLI-16-18155).doc]

中共中央办公厅、国务院办公厅关于转发《国务院妇女儿童工作委员会关于三年来我国妇女儿童工作情况和“九五”期间工作任务的报告》的通知

**发布部门：** [中共中央办公厅](https://www.pkulaw.com/law/chl?Aggs.IssueDepartment=a03) [国务院办公厅](https://www.pkulaw.com/law/chl?Aggs.IssueDepartment=601) [机构沿革](javascript:void(0))

**发文字号：**厅字〔1997〕12号

**发布日期：**1997.04.26

**实施日期：**1997.04.26

**时效性：** [现行有效](https://www.pkulaw.com/law/chl?Aggs.TimelinessDic=01)

**效力级别：** [党内法规制度](https://www.pkulaw.com/law/chl?Aggs.EffectivenessDic=XR12)

**法规类别：** [老少妇幼残保护](https://www.pkulaw.com/law/chl?Aggs.Category=01604)

　　　　　　　　　　中共中央办公厅　国务院办公厅关于
　　　　　　　　转发《国务院妇女儿童工作委员会关于三年来
　　　　我国妇女儿童工作情况和“九五”期间工作任务的报告》的通知
　　　　　　（厅字〔1997〕12号　1997年4月26日）

　　《国务院妇女儿童工作委员会关于三年来我国妇女儿童工作情况和“九五”期间工作任务的报告》，已经党中央、国务院领导同志同意，现转发给你们，请结合本地区、本部门实际情况，认真贯彻执行。
　　　　　　　　　　　　　　　　　　　中共中央办公厅　国务院办公厅
　　　　　　　　　　　　　　　　　　　　　　　　　1997年4月26日
　　　　　　　　　　　国务院妇女儿童工作委员会关于
　　　　 三年来我国妇女儿童工作情况和“九五”期间工作任务的报告
党中央、国务院：
　　国务院妇女儿童工作委员会成立三年来，在党中央、国务院的领导下，在促进妇女儿童事业发展和保障妇女儿童合法权益方面做了大量工作，取得了明显成效。现将三年来我国妇女儿童工作情况和“九五”期间工作任务报告如下：
　　**一、**三年来的主要工作和基本经验
　　三年来，我国妇女儿童事业有了新的进展。国务院妇女儿童工作委员会主要做了以下六项工作；第一，起草并报请国务院颁布了《[中国妇女发展纲要](https://www.pkulaw.com/chl/33670a50094f0c40bdfb.html)》（以下简称[《妇女纲要》](https://www.pkulaw.com/chl/33670a50094f0c40bdfb.html)），推动各地区、各部门制定了实施规划。目前，各省、自治区、直辖市都颁布了本地区妇女发展规划，一些市、县也制定了妇女发展规划；劳动部、农业部、国家计生委、卫生部、人事部已先后制定了实施[《妇女纲要》](https://www.pkulaw.com/chl/33670a50094f0c40bdfb.html)的规划。第二，进一步推动了《九十年代中国儿童发展规划纲要》（以下简称《儿童纲要》）的实施，基本实现了九十年代的中期目标，向联合国提交了《九十年代中期中国儿童发展状况报告》，受到好评。第三，就当前妇女儿童发展中的突出问题，组织、协调政府有关部门办了一些实事。针对下岗女职工增多的问题，推动有关部门研究解决办法；积极推进生育保险费用社会统筹；针对一些外商投资企业、私营企业侵犯女职工合法权益的问题，促进地方政府和有关部门查处并加以解决；针对卖淫嫖娼屡禁不止的问题，多次协同有关部门研究并采取措施加以遏制；针对西方对我国儿童福利院的攻击以及我国在保护孤儿、制止弃婴工作中存在的问题，提出加强这方面工作的意见，明确各有关部门的责任。第四，推动了《[妇女权益保障法](https://www.pkulaw.com/chl/dff2eefbddf155b3bdfb.html)》和《[未成年人保护法](https://www.pkulaw.com/chl/f676777bde217880bdfb.html)》的实施和执法检查工作。第五，进一步推动各地区建立健全妇女儿童工作机构。目前，各省、自治区、直辖市和计划单列市都成立了妇女儿童工作机构，由政府负责同志担任主任，办公室设在妇联。全国多数市、县也都建立了妇女儿童工作机构。第六，筹备和参与第四次世界妇女大会的有关工作，推动了我国妇女儿童事业的发展。
　　三年来，全国在贯彻实施《[妇女权益保障法](https://www.pkulaw.com/chl/dff2eefbddf155b3bdfb.html)》、《[未成年人保护法](https://www.pkulaw.com/chl/f676777bde217880bdfb.html)》和[《妇女纲要》](https://www.pkulaw.com/chl/33670a50094f0c40bdfb.html)、《儿童纲要》中，积累了一些经验，初步探索出一条适合我国国情的妇女儿童工作路子。这就是，党委、政府重视，政府统筹协调，发挥人大、政协作用，有关部门齐抓共管；在各级政府建立妇女儿童工作机构；广泛动员社会各界积极参与，充分依靠广大群众；抓住妇女儿童发展中的重点和难点问题加以突破，实行分类指导，建立监测评估机制，推动实施《[妇女权益保障法](https://www.pkulaw.com/chl/dff2eefbddf155b3bdfb.html)》、《[未成年人保护法](https://www.pkulaw.com/chl/f676777bde217880bdfb.html)》和[《妇女纲要》](https://www.pkulaw.com/chl/33670a50094f0c40bdfb.html)、《儿童纲要》。实践证明，各级政府妇女儿童工作委员会的建立，使各级政府更好地承担起发展妇女儿童事业、维护妇女儿童合法权益的责任，加强了有关部门之间、政府与群众团体之间的密切协作，促使妇女儿童事业得以整体推进。

　　**二、**当前妇女儿童工作的形势
　　目前，我国妇女儿童工作的环境、条件以及妇女儿童工作的发展状况都有了长足的进步。妇女的参政状况、劳动就业与社会保障程度、受教育程度、健康状况等都达到了历史最好水平；儿童发展的绝大多数指标已达到或超过九十年代中期目标的要求，受到国际社会赞誉。但我国的妇女儿童事业还存在着不少困难和亟待解决的问题。城乡之间、地区之间妇女儿童工作发展水平极不平衡，农村特别是贫困地区实施[《妇女纲要》](https://www.pkulaw.com/chl/33670a50094f0c40bdfb.html)、《儿童纲要》的难度较大；下岗女职工人数呈增多趋势，女性再就业的难度很大；在相当一部分外商投资企业、私营企业和乡镇企业中，女职工的劳动保护不落实，有的女职工合法权益受到严重侵害；流动人口中妇女儿童的困难和问题较多，尚未引起足够重视；弃婴和流浪儿的数量仍在增加，一些地方婴儿出生性别比例失调；拐卖、绑架妇女儿童的犯罪活动和卖淫嫖娼、色情服务等社会丑恶现象屡禁不止；妇女遭受暴力侵犯屡有发生；招工、招生、分房和承包责任田、口粮田以及划分宅基地等方面的男女不平等现象还普遍存在；女性参政的比例仍然偏低。
　　产生上述问题的原因是多方面的，既受到经济和社会发展水平的制约，也受到传统观念及经济和社会生活重大变动的影响，而体制、法律、政策、管理的不完善，忽视精神文明建设，以及一些人道德失范、见利忘义、侵犯妇女儿童权利，则加重了解决妇女儿童问题的难度。因此，要充分认识到促进妇女儿童发展和保障妇女儿童权益是一项长期、艰巨、复杂的任务，要以坚强的决心和毅力把这项工作搞好。

　　**三、**“九五”期间我国妇女儿童工作的任务
　　根据党的十四届五中、六中全会精神以及我国妇女儿童工作的实际，“九五”期间妇女儿童工作总的指导思想是：以马列主义、毛泽东思想和邓小平建设有中国特色社会主义理论为指导，坚持党的基本路线和基本方针，认真贯彻落实中央对妇女儿童工作的指示精神，全面执行《[妇女权益保障法](https://www.pkulaw.com/chl/dff2eefbddf155b3bdfb.html)》和《[未成年人保护法](https://www.pkulaw.com/chl/f676777bde217880bdfb.html)》，积极实施[《妇女纲要》](https://www.pkulaw.com/chl/33670a50094f0c40bdfb.html)和《儿童纲要》，把农村特别是贫困地区的妇女儿童工作作为重点，努力使妇女儿童工作有一个大的发展，更好地为经济建设和社会全面进步服务。根据上述指导思想，“九五”时期妇女儿童工作的主要任务是：
　　1．各级党委和政府要进一步加强对妇女儿童工作的领导，树立马克思主义妇女观，坚定不移地贯彻男女平等的原则。我国妇女儿童占总人口的70％。是否把妇女儿童工作摆到党委和政府工作的重要位置，是衡量领导同志是否具有远见卓识的重要标志之一。各级党委和政府要把妇女儿童事业纳入国民经济和社会发展的总体规划，采取有力措施，进一步促进妇女儿童工作的发展。
　　2．把实施[《妇女纲要》](https://www.pkulaw.com/chl/33670a50094f0c40bdfb.html)和《儿童纲要》作为妇女儿童工作的中心任务，保证到2000年两个纲要规定的各项目标得以实现。各级政府要认真实行目标管理责任制，把任务分解到责任单位，分级、分项、分年度制定计划和措施，加以落实。要加强监测评估工作，制定和完善两个纲要的监测统计指标体系和评估方案。各级政府都要随着财政收入的增长逐步增加实施两个纲要所需的经费投入。
　　3．在妇女儿童工作中，切实把社会主义精神文明建设提到更加突出的地位。要把《[妇女权益保障法](https://www.pkulaw.com/chl/dff2eefbddf155b3bdfb.html)》、《[未成年人保护法](https://www.pkulaw.com/chl/f676777bde217880bdfb.html)》纳入“三五”普法规划，进一步加大宣传这两部法律和两个纲要的力度，在全社会形成尊重妇女儿童、保护妇女儿童权益的良好社会风尚。要把提高妇女的整体素质作为加强精神文明建设的一项重要任务，努力提高她们的思想道德素质和科学文化素质，培养有理想、有道德、有文化、有纪律和自尊、自信、自立、自强的女性。要做好培养女干部和发展女党员的工作。要鼓励广大妇女积极投身社会主义现代化建设，引导她们树立建设有中国特色社会主义的共同理想和正确的世界观、人生观、价值观，树立文明、健康、科学的婚育观，弘扬社会主义的社会公德、职业道德、家庭美德。要大力加强少年儿童的思想道德教育。要特别关心处于困难条件下的少年儿童（包括残疾儿童、离异家庭的儿童、孤儿和流浪儿等），为他们的健康成长创造良好条件。
　　4．把做好农村的妇女儿童工作作为实施两个纲要的重点。做好农村的妇女儿童工作，对提高农村人口素质，促进农村经济和社会发展具有重要的战略意义。在农村，工作的难点在贫困地区。各级党委和政府以及有关部门要把扶助贫困地区实施两个纲要的工作纳入本地区、本部门的扶贫开发总体规划，采取有效措施从人、财、物等方面加以保证。农村特别是贫困地区实施两个纲要，主要应加强农村基层组织建设，发挥妇代会的作用，组织广大妇女积极参加农村各业生产，开展“双学双比”活动，努力学习文化科学技术，尽快改变农村妇女的贫困状况；同时要抓好教育、卫生和计划生育工作，改善妇女儿童的生活质量和生存环境。妇女在贫困家庭中最困苦、承担压力最大，实现本世纪末解决我国贫困人口温饱问题的战略目标，需要对妇女脱贫给予特殊的关注和扶持。要积极推动“巾帼扶贫行动”和“幸福工程－－救助贫困母亲行动”的开展，促进扶贫开发与计划生育相结合。各级妇女儿童工作委员会要加强对农村特别是贫困地区的指导和帮助，深入调查研究，及时总结经验，研究存在的问题和解决的办法，推动农村妇女儿童工作进一步向前发展。
　　5．深入实际，着力研究解决妇女儿童发展中出现的新情况、新问题，为妇女儿童办实事。当前需要研究解决的问题主要是：（1）妇女就业问题。要严格执法，坚持在劳动权益方面实现男女平等。要加强对失业、下岗女职工的教育和培训，帮助她们树立正确的择业观念，提高就业技能。要坚持多渠道就业的方针，为妇女创造更多的就业机会。（2）女职工劳动保护问题。要进一步健全劳动监察法规，加强劳动监察力量，加大劳动监察力度，认真开展巡视监察、举报专查等执法检查活动，严肃查处违反女职工劳动保护法律、法规的案件和行为。加强劳动合同管理，规范劳动关系。加大女职工生育保险制度改革的力度，继续扩大生育保险覆盖面，在全国基本建立起市（县）级生育保险费用社会统筹制度。（3）“打拐”、“禁娼”问题。拐卖妇女儿童犯罪活动和卖淫嫖娼的丑恶现象是与社会主义制度不相容的，对社会风气和妇女儿童危害极大。各级政法、工商、旅游部门和文化娱乐及其他服务行业要各负其责，齐抓共管，进一步加大“打拐”、“禁娼”的力度，狠抓大案要案的查处。（4）孤残儿童福利事业问题。要重视儿童福利院建设，做到布局合理，保证儿童生活不低于当地人均生活水平。积极支持和引导社会各界和公民个人依法兴办社会儿童慈善事业，提倡儿童福利机构公办、民办并举。鼓励群众依法收养儿童。（5）弃婴和流浪儿的救助保护问题。要宣传弃婴是违法犯罪行为，有悖于社会公德和家庭美德。要加强计划生育管理，防止和减少弃婴现象的发生。要认真贯彻《[母婴保健法](https://www.pkulaw.com/chl/ea633acd04298a40bdfb.html)》，最大限度地避免和减少先天性残疾婴儿的出生。要建立健全社会保障制度，妥善做好弃婴的安置工作。要从实际出发，研究解决群众自愿收养弃婴中亟待解决的问题。要认真贯彻落实《中共中央办公厅、国务院办公厅关于转发＜[中央社会治安综合治理委员会关于加强流动人口管理工作的意见＞的通知](https://www.pkulaw.com/chl/d992207ee21ea528bdfb.html)》（厅字〔1995〕42号）精神，进一步加强流浪少年儿童保护教育中心建设，切实做好流浪儿的救助保护工作。上述问题都涉及妇女儿童的基本权益、基本生活保障，必须以全心全意为人民服务的精神为她们排忧解难。各级妇女儿童工作委员会要抓住本地区、本部门最迫切需要解决的问题，认真调查研究，做好工作，力争在“九五”期间使这些问题得到较好解决。
　　6．进一步发挥各级妇女儿童工作委员会作用。妇女儿童工作委员会要突出议事和协调两方面的作用，组织、协调、推动有关部门和团体一起研究解决妇女儿童工作中的重大问题，共同实施《[妇女权益保障法](https://www.pkulaw.com/chl/dff2eefbddf155b3bdfb.html)》、《未成年人保障法》和[《妇女纲要》](https://www.pkulaw.com/chl/33670a50094f0c40bdfb.html)、《儿童纲要》。妇女儿童工作委员会每年都要深入调查研究几个重大问题，推动社会一起来解决。
　　以上报告如无不妥，建议批转各地区、各部门贯彻执行。
　　　　　　　　　　　　　　　　　　　　　国务院妇女儿童工作委员会
　　　　　　　　　　　　　　　　　　　　　　　　　1997年3月26日

© 北大法宝：（[www.pkulaw.com](https://www.pkulaw.com/)) 专业提供法律信息、法学知识和法律软件领域各类解决方案。北大法宝为您提供丰富的参考资料，正式引用法规条文时请与标准文本核对。
欢迎查看所有[产品和服务](https://www.pkulaw.com/helps/2.html)。法宝快讯：[如何快速找到您需要的检索结果？](https://www.pkulaw.com/helps/70.html)[法宝](https://www.pkulaw.com/helps/69.html)V6有何新特色？

扫描二维码阅读原文

**原文链接：**<https://www.pkulaw.com/chl/62c73e403b470fabbdfb.html>
